# Morphology-Dependent Behavior of PVDF/ZnO Composites: Their Fabrication and Application in Pressure Sensors

**DOI:** 10.3390/s25092936

**Published:** 2025-05-07

**Authors:** Binbin Zhang, Wenhui Zhang, Wei Luo, Zhijie Liang, Yan Hong, Jianhui Li, Guoyun Zhou, Wei He

**Affiliations:** 1 School of Materials and Energy, University of Electronic Science and Technology of China, Chengdu 611731, China; zhang0001234@163.com (B.Z.); 202221030230@std.uestc.edu.cn (W.Z.); hongyan@uestc.edu.cn (Y.H.); he_wei@uestc.edu.cn (W.H.); 2 Beijing Spacecraft, China Academy of Space Technology, Beijing 100094, China; 3 School of Integrated Circuits, Huazhong University of Science and Technology, Wuhan 430074, China; 4 Jiangxi Institute of Electronic Circuit, Pingxiang 337009, China; liangzj0214@163.com; 5 Suining Ruijiexing Technology Co., Ltd., Suining 629001, China; ljh@rjx-pcb.com

**Keywords:** pressure sensors, zinc oxide, PVDF, composite materials

## Abstract

This study investigated the impact of zinc oxide’s (ZnO’s) morphology on the piezoelectric performance of polyvinylidene fluoride (PVDF) composites for flexible sensors. Rod-like (NR) and sheet-like (NS) ZnO nanoparticles were synthesized via hydrothermal methods and incorporated into PVDF through direct ink writing (DIW). The structural analyses confirmed the successful formation of wurtzite ZnO and enhanced β-phase content in the PVDF/ZnO composites. At a degree of 15 wt% loading, the ZnO-NS nanoparticles achieved the highest β-phase fraction (81.3%) in PVDF due to their high specific surface area, facilitating dipole alignment and strain-induced crystallization. The optimized PVDF/ZnO-NS-15 sensor demonstrated superior piezoelectric outputs (4.75 V, 140 mV/N sensitivity) under a 27 N force, outperforming its ZnO-NR counterparts (3.84 V, 100 mV/N). The cyclic tests revealed exceptional durability (<5% signal attenuation after 1000 impacts) and a rapid response (<100 ms). The application trials validated their real-time motion-monitoring capabilities, including finger joint flexion detection. This work highlights the morphology-dependent interfacial polarization as a critical factor for high-performance flexible sensors, offering a scalable DIW-based strategy for wearable electronics.

## 1. Introduction

In recent decades, polyvinylidene fluoride (PVDF) has emerged as an ideal substrate for flexible electronic devices due to its unique piezoelectricity, high mechanical flexibility, and chemical inertness [1,2,3]. Compared to conventional inorganic piezoelectric materials, the lightweight nature and bendability of PVDF make the corresponding substrate compatible with human skin and curved structures, demonstrating broad application prospects for wearable health monitoring and robotic tactile sensing [4,5]. Generally, PVDF sustains particular crystalline phases with varied molecular orientations (α (TGTG’), β (TTTT), γ, δ, and ε), which form under distinct conditions and interconvert via thermal, electric field, or mechanical stretching treatments [6,7]. It is well known that the β-phase, characterized by an all-trans (TTTT) conformation, serves as the electroactive polar phase [8]. Enhancing the β-phase content is, therefore, one of the major approaches to significantly improving the piezoelectric performance of PVDF [9].

To optimize the electroactive phase of PVDF, researchers have explored various techniques, including mechanical stretching [10], electrospinning [11], high-voltage poling [12], direct ink writing (DIW) [13], and nanofiller incorporation [14]. The incorporation of nanomaterials presents a cost-effective and efficient strategy. Recent studies have developed diverse synthesis methods involving nanoparticles to enhance the crystalline orientation of PVDF. Piezoelectric inorganic particles, including barium titanate (BTO) [15], lead zirconate titanate (PZT) [16], and zinc oxide (ZnO) [17], have been introduced into PVDF matrixes. For instance, Yang et al. fabricated composite membranes including polydopamine, BTO, and PVDF via solution casting, achieving a sensitivity of 64.2 mV/N [12]. Guo et al. designed a self-powered PVDF composite piezoelectric sensor with a baklava-like layered crystalline structure, where mechanical compression was applied post-solution casting to realign the PVDF molecular chains and enhance the β-phase content, reaching an exceptional sensitivity (6.38 mV/N), an ultrafast response time (21 ms), and high feasibility for the real-time monitoring of table tennis training with scalable motion analysis [18].

Among the polymer–ceramic composites, ZnO-based systems have garnered extensive attention due to their superior biocompatibility, pyroelectricity, flexibility, and facile synthesis [19,20]. Arjun et al. fabricated PVDF composite films with ZnO, TiO_2_, and SiO_2_ nanoparticles, reporting a maximum sensitivity of 103 mV/N for the ZnO-doped sensors [21]. Chen et al. employed electric field-assisted DIW to prepare PVDF/ZnO films, where the aligned PVDF molecular chains under electric fields enhanced the β-phase crystallinity. The optimal ZnO loading yielded an output voltage of 351 mV [13]. These works have demonstrated the considerable potential of composite PVDF sensors incorporating ZnO nanoparticles. It is well known that the morphology of the doped nanoparticles is a crucial factor affecting the performance of polymer–particle composites [22,23]. However, discussions on the relation between the morphologic features of ZnO nanoparticles and the sensing performance of PVDF/ZnO composites remain scarce.

In this work, high-performance sensors were fabricated by doping PVDF with ZnO of varied morphologies via direct ink writing. The materials were subjected to a morphological characterization and compositional analysis using scanning electron microscopy (SEM), Fourier-transform infrared spectroscopy (FTIR), and X-ray diffraction (XRD) analysis. The piezoelectric properties of the films were evaluated, and the piezoelectric responses of the sensors were characterized through cyclic impact testing.

## 2. Materials and Methods

### 2.1. Materials

PVDF powder (average Mw ≈ 534,000) was sourced from Sigma-Aldrich (Shanghai, China). Other chemicals, including N,N-dimethylformamide (DMF, ≥99.5%), zinc acetate dihydrate (C_4_H_6_O_4_Zn·2H_2_O, ≥99%), sodium hydroxide (NaOH, ≥96%), zinc nitrate hexahydrate (Zn(NO_3_)_2_·6H_2_O, ≥99%), ethylenediamine monohydrate (C_2_H_8_N_2_·H_2_O, 98%) and ethanolamine (C_2_H_7_NO, 99%) were procured from Aladdin Biochemical Technology Co., Ltd. (Shanghai, China).

### 2.2. Synthesis of ZnO with Controlled Morphologies

**Figure 1:**
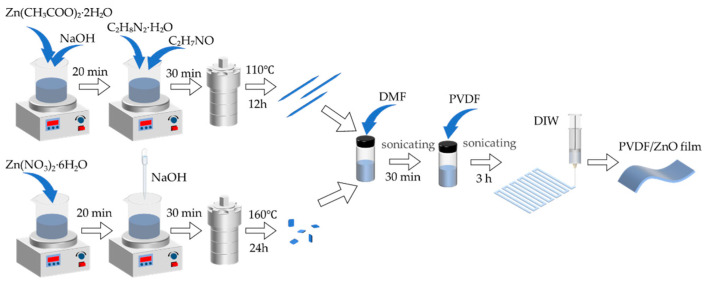
Schematic flowchart illustrating preparation of composite sensing film.

The rod-like ZnO nanostructures were synthesized via a hydrothermal method (Figure 1). In a typical procedure, 1 g of Zn(CH_3_COO)_2_·2H_2_O and 1 g of NaOH were dissolved in 15 mL of deionized water under magnetic stirring for 20 min. Subsequently, 15 mL of C_2_H_8_N_2_·H_2_O and 15 mL of C_2_H_7_NO were added to the mixture under vigorous stirring for 30 min. The homogeneous solution was transferred into a 100 mL Teflon-lined stainless steel autoclave and maintained at 110 °C for 12 h in an oven. After natural cooling to room temperature, the precipitate was collected by centrifugation (4000 rpm, 15 min), washed repeatedly with deionized water until neutral pH was achieved, and dried at 60 °C for 12 h. The resulting white powder was ground into fine rod-shaped ZnO particles for subsequent use.

The sheet-like ZnO was prepared through a hydrothermal protocol. Initially, 2.25 g Zn(NO_3_)_2·_6H_2_O was dissolved in 45 mL of deionized water under continuous magnetic stirring. NaOH solution was then dropwise added to adjust the pH to 13, forming a milky suspension. After 30 min of homogenization, the mixture was transferred into a 100 mL Teflon-lined autoclave and heated at 160 °C for 24 h. The cooled product underwent identical centrifugation, washing, and drying procedures as described for rod-shaped ZnO, ultimately yielding thin ZnO nanosheets.

### 2.3. Preparation of PVDF/ZnO Composite Films

The total mass of the precursor solution was fixed at 10 g. ZnO with distinct morphologies—nanorods (NRs) and nanosheets (NSs)—were weighed into 0.1, 0.2, 0.3, and 0.4 g samples, respectively, and dispersed into 7.9, 7.8, 7.7, and 7.6 g of DMF under ambient conditions via 1 h ultrasonication. Subsequently, 2 g of PVDF was introduced into each mixture, followed by additional 3 h ultrasonication to ensure complete dissolution. The homogeneous solutions were degassed through 0.5 h static settling, yielding PVDF/ZnO printing inks with ZnO mass fractions of 5 wt%, 10 wt%, 15 wt%, and 20 wt%. A pure PVDF control solution was separately prepared by mixing 2 g PVDF with 8 g DMF. The nanocomposites were designated as PVDF/ZnO-NRs(nanorods)-X and PVDF/ZnO-NSs(nanosheets)-X, where ‘X’ denotes the ZnO content (5, 10, 15, or 20 wt%).

The prepared ink was loaded into a syringe equipped with a nozzle positioned 2 mm above a glass substrate preheated to 90 °C. The printing parameters were configured with extrusion multiplier of 0.4, layer height of 0.15 mm, and nozzle travel speed of 5 mm/s. The extrusion line width was adjusted to 1.1 mm to compensate for the oozing effect, while a double-layer skirt structure with 2 mm spacing was implemented to optimize bed adhesion stability. For preventing stringing during nozzle homing, retraction settings were configured with 2 mm retraction distance and 30 mm/s retraction speed. Precise material control was achieved through the combination of a 0.41 mm nozzle diameter and 40% internal infill density. A 4 cm × 4 cm multilayer film was fabricated through layer-by-layer deposition at a printing speed of 5 mm/s, with alternating X- and Y-axis toolpaths to enhance interlayer adhesion. Following two-layer deposition, the film was oven-dried, achieving a final thickness of approximately 100 μm.

### 2.4. Preparation of PVDF/ZnO-Based Sensors

**Figure 2:**
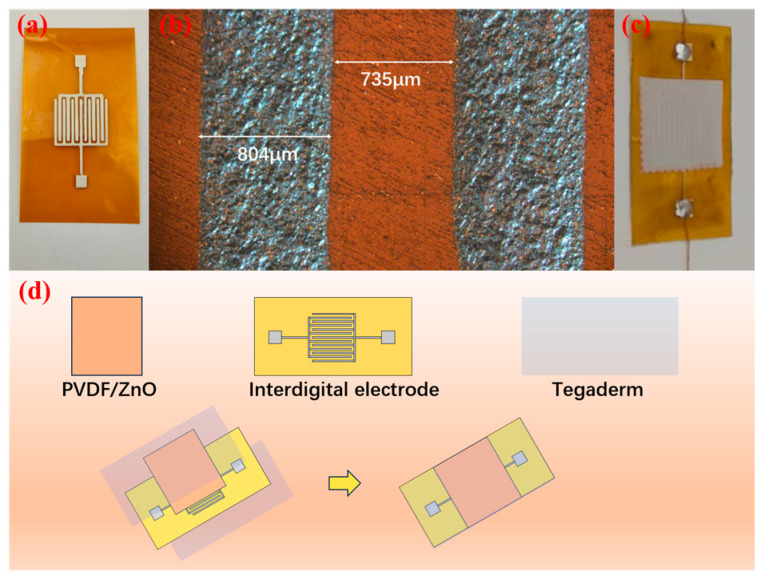
Microscopic images of the flexible sliver electrode from (**a**) top view and (**b**) magnified pattern; (**c**) microscopic image of the assembled flexible pressure sensor; and (**d**) schematic illustration of the fabrication process for the PVDF/ZnO flexible piezoelectric sensor.

For the fabrication of interdigitated electrodes for piezoelectric sensors, silver paste was prepared with the following specifications: particle size ≤ 8 μm, viscosity of 80–330 Pa·s, and solid content of 65–80%. High-purity silver paste was injected into a 10 mL syringe and dispensed onto a polyimide (PI) substrate through a direct-write process at a controlled printing speed of 5 mm/s. The patterned electrodes were thermally cured at 80 °C for 30 min, followed by wire bonding to the contact pads of the interdigitated electrodes. The printed features exhibited a line width of 803 μm and a line spacing of 735 μm, as illustrated in Figure 2a,b. Subsequently, the composite film was cut into 2 cm × 2 cm sections, and silver interdigitated electrodes were laminated onto the film surface. To ensure environmental stability, the pressure sensor was encapsulated with flexible medical-grade adhesive tape, providing protection against contamination and corrosion. The finalized sensor prototype is shown in Figure 2c. The schematic preparation process is illustrated in Figure 2d.

### 2.5. Characterizations

The morphologies of ZnO nanostructures and composite films were characterized by SEM (Hitachi, SU5000, Tokyo, Japan). Fourier-transform infrared (FT-IR) spectroscopy of the composite films was performed on a Shimadzu IRTracer 100 spectrometer (Tokyo, Japan). Crystalline structures were analyzed via XRD (Shimadzu, XRD-7000, Tokyo, Japan) with Cu-Kα radiation (λ = 1.5406 Å).

**Figure 3:**
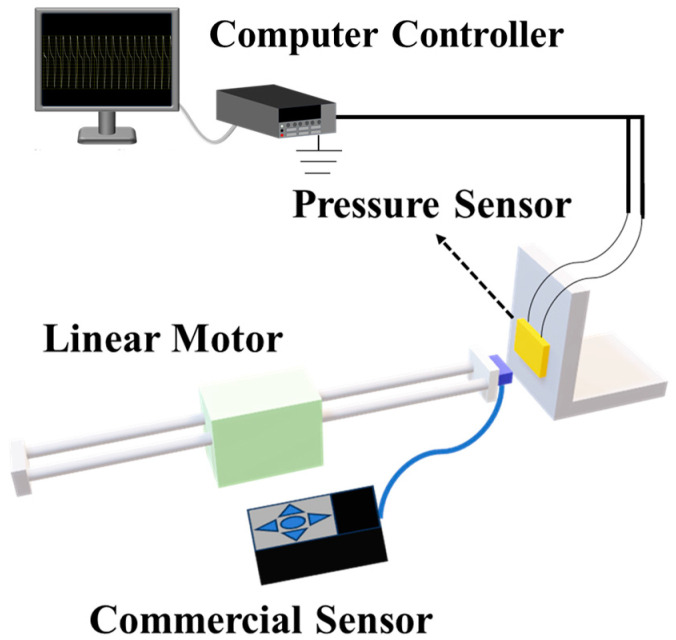
Schematic diagram of the piezoelectric performance test system.

As illustrated in Figure 3, the testing system comprised an NTIAG HS01-37 × 166 linear motor (Lake Geneva, WI, USA) to generate programmable external stimuli. Electrical properties were measured using a Keithley 6514 high-resistance electrometer (Shanghai, China). During the testing process, the commercial sensor was mounted on the front end of the stepper motor. Subsequently, the prepared composite film sensor and linear motor were co-mounted onto an acrylic bracket at equivalent height. The leads from the film were connected to the positive and negative terminals of a 6514 electrometer, respectively, with the instrument grounded to eliminate electromagnetic interference. For force calibration, a commercial reference sensor (model ZPS-Z2S-DPU-50N, AIGU Technologies, Hong Kong, China) was utilized for measurement, with the applied force magnitude determined by averaging multiple measurement datasets.

## 3. Results

### 3.1. Characterization of the Synthesized ZnO Particles

**Figure 4:**
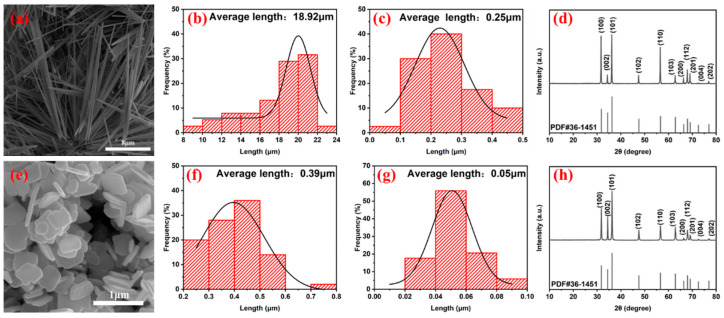
(**a**) SEM image, (**b**) particle size distribution for the longitudinal axis, (**c**) particle size distribution for the transverse axis, and (**d**) XRD pattern of ZnO-NRs. (**e**) SEM image, (**f**) particle in-plane size distribution, (**g**) thickness distribution, and (**h**) XRD patterns of ZnO-NSs.

The morphology and crystal structure of the synthesized ZnO nanoparticles were investigated through SEM and XRD measurements, respectively (Figure 4). The SEM images in Figure 4a–c reveal that the rod-like ZnO exhibited an average length of 18.92 μm, an average diameter of 0.25 μm, and a length-to-diameter ratio as high as 75. The SEM images in Figure 4e–g show that the plate-like ZnO had an average particle size of 0.39 μm and an average thickness of 0.05 μm.

The XRD pattern of the ZnO, as shown in Figure 4d,h, demonstrates that the acquired diffraction peaks of the synthesized ZnO match well with those of a standard JCPDS card (PDF#36-1451), without any impurity peaks observed. The profound XRD features indicate that the product has a wurtzite hexagonal structure, which is a representative structure for a ZnO nanoparticle synthesized through a hydrothermal route [24]. The prominent diffraction peaks at the (100), (101), and (110) planes suggest that the synthesized ZnO maintains good crystallinity.

### 3.2. Characterization and Application of the Piezoelectric Composite Films

**Figure 5:**
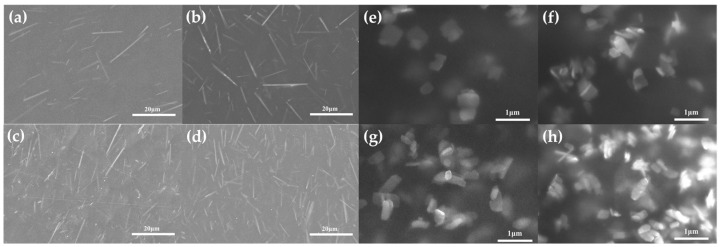
The SEM images of the PVDF/ZnO-NRs-X with a varied content of ZnO-NRs of (**a**) 5 wt%, (**b**) 10 wt%, (**c**) 15 wt%, and (**d**) 20 wt%. The SEM images of the PVDF/ZnO-NSs-X with a varied content of ZnO-NSs of (**e**) 5 wt%, (**f**) 10 wt%, (**g**) 15 wt%, and (**h**) 20 wt%.

Figure 5 presents the microstructural morphology images of the ZnO-incorporated composite films with varied filler loadings. Notably, the composite films exhibit defect-free interfaces without observable cracks or interfacial voids, even at elevated ZnO concentrations. The SEM images of the composite films confirm the homogeneous dispersion of both the rod-like and sheet-shaped ZnO particles within the PVDF matrix at low concentrations (Figure 5a,b,e,f). A minor agglomeration occurs at higher loading levels (Figure 5c,d,g,h).

**Figure 6:**
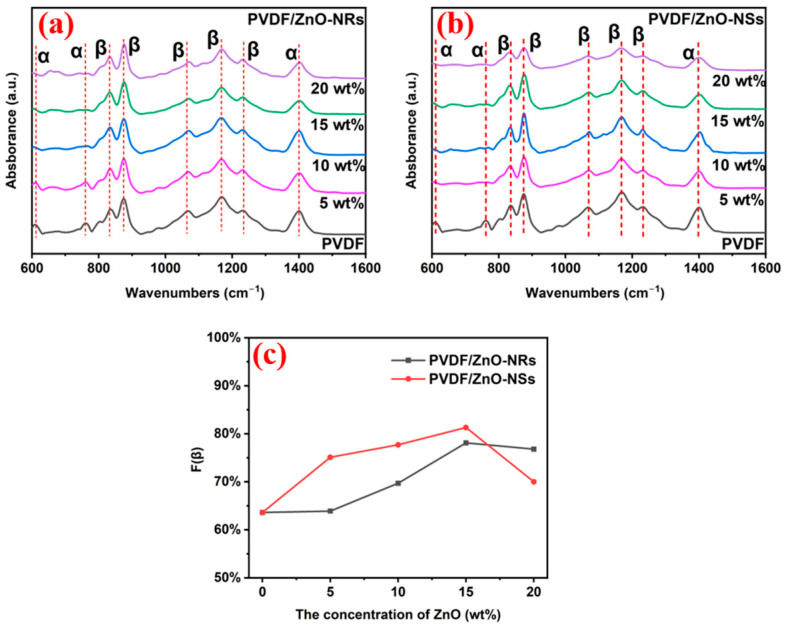
(**a**) FT-IR spectra of PVDF/ZnO-NRs-X; (**b**) FT-IR spectra of PVDF/ZnO-NSs-X; and (**c**) β-phase content of samples.

The crystalline phases of the PVDF films with varied ZnO morphologies and loading ratios were systematically investigated through FTIR, as shown in Figure 6. Distinct absorption bands corresponding to both the non-polar α-phase and polar β-phase were identified, confirming the coexistence of multiple crystalline forms in the DIW-fabricated PVDF composites.

In Figure 6a,b, the α-phase exhibits characteristic peaks at 614, 763, 975, and 1209 cm^−1^, while the β-phase manifests vibrational signatures at 840, 874, 1070, 1167, and 1274 cm^−1^. The bands at 614 and 764 cm^−1^ were assigned to the out-of-plane bending/rocking vibrations of the CF_2_ groups and the skeletal rocking vibrations of the PVDF chains, respectively. The prominent β-phase absorption at 840 cm^−1^ originated from the in-plane rocking vibration of the H-C-H bonds coupled with the symmetric stretching of the F-C-F groups. The strong peak at 874 cm^−1^ arose from the synergistic interaction of the CH_2_ in-plane rocking and the CF_2_ asymmetric stretching modes [25]. The 1070 cm^−1^ band corresponds to antisymmetric C-C stretching vibrations. The 1167 and 1230 cm^−1^ peaks were attributed to C-H-C wagging vibrations. The 1400 cm^−1^ feature indicates C-F stretching modes. These features all imply the coexistence of α- and β-phases within the PVDF matrix.

As shown in Figure 6, the addition of ZnO to the PVDF composite films resulted in a significant decrease in the absorption intensity of the α-phase characteristic peaks and a notable increase in the characteristic peak intensities of the β-phase. This divergent trend for the α- and β-phases indicates that the introduced ZnO promoted the transformation of the α-phase into the β-phase. The primary reason for this phenomenon was due to the negative surface charge of the ZnO fillers. When the ZnO fillers were introduced into the PVDF matrix, interactions occurred between the -CH_2_ dipoles of the PVDF and the negative surface charge of the ZnO fillers, leading to the formation of TTTT structures in the PVDF [26]. Additionally, during the extrusion process, the PVDF was subjected to stretching forces, which induced the preferential orientation and alignment of the PVDF chains, resulting in the formation of more β-phase structures.

The β-phase content (*F*(*β*)) was quantitatively estimated using the Lambert–Beer relationship:

$$F\left(\beta \right)=\frac{A(\beta )}{\frac{K_{\beta \;}}{K_{\alpha }}A(\alpha )+A(\beta )}$$

The absorption intensities at 763 cm^−1^ and 840 cm^−1^, denoted as *A*(*α*) and *A*(*β*), were used to quantify the relative contributions of the α-phase and β-phase in the ZnO-doped PVDF composite films. *K_β_*/*K_α_*, representing the ratio of the β-phase absorption coefficient (7.7 × 10^4^ cm^2^/mol) to the α-phase absorption coefficient (6.1 × 10^4^ cm^2^/mol), was calculated as 1.26. The values of *F*(*β*) for the samples are shown in Figure 6c. The results demonstrate that the ZnO-NRs and ZnO-NSs-doped films exhibited the highest β-phase content of 78.1% and 81.3%, respectively, at a ZnO doping content of 15 wt%. However, for the films with a ZnO content exceeding 15 wt%, a decreasing trend in the β-phase content was observed. This decrement in the β-phase content was attributed to the aggregation of the ZnO particles at higher doping levels, which hindered crystallization and ultimately affected the β-phase content of the films.

Compared to the composites with ZnO-NRs, the enhanced β-phase formation observed in the PVDF composites incorporating ZnO-NSs was primarily attributed to the higher specific surface area (SSA) of the nanosheets. The larger contact area facilitated stronger dipole coupling between the surface of the ZnO nanosheets and the -CH_2_ groups in the PVDF chains. The enhanced interfacial polarization promoted the alignment of the molecular dipoles into the electroactive TTTT conformational characteristics. Additionally, the planar structure of the nanosheets induced localized stress concentrations during mechanical deformation, serving as nucleation sites for β-phase crystallization through strain-induced molecular rearrangements. This mechanism synergized with the enhanced surface charge interactions, achieving an exceptional β-phase content of 81.3% in the composite matrix with the ZnO-NSs, representing a significant improvement from the 78.1% β-phase content with the ZnO-NRs fillers.

**Figure 7:**
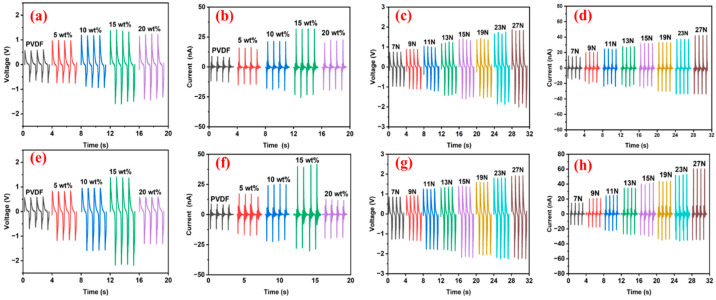
Output (**a**) voltage and (**b**) current of composite sensors with varying ZnO-NRs content under applied force of 15 N. Output (**c**) voltage and (**d**) current under different applied pressures. Output (**e**) voltage and (**f**) current of composite sensors with varying ZnO-NSs content under applied force of 15 N. Output (**g**) voltage and (**h**) current under different applied pressures.

The PVDF/ZnO composite sensors exhibited alternating current characteristics in their electrical signal outputs (Figure 7). When pure PVDF served as the sensing layer, the pressure sensor demonstrated a peak-to-peak output voltage of 1.26 V, with a nanoampere-level current (Figure 7a,b,e,f). ZnO doping significantly enhanced both the voltage and current outputs, yet excessive ZnO concentrations (>15 wt%) caused signal attenuation due to the nanoparticle agglomeration-induced deterioration of the electromechanical coupling efficiency, as evidenced by the FTIR analyses.

The optimized ZnO content was determined to be 15 wt%, as the PVDF/ZnO-NRs-15 achieved a maximum value for the peak-to-peak output voltage of 3.0 V (138% enhancement vs. pure PVDF), while the PVDF/ZnO-NSs-15 reached 3.56 V (183% enhancement), as plotted in Figure 7a,e. The superior performance of the ZnO-NSs resulted from the higher SSA, which promoted interfacial polarization under mechanical stress, thereby generating enhanced piezoelectric charges. As a result, the PVDF/ZnO-NRs-15 and PVDF/ZnO-NSs-15 were selected for subsequent piezoelectric evaluations.

The output performance of the pressure sensors exhibited a significant dependence on the applied external forces. To systematically investigate this pressure–response relationship, the voltage and current outputs of the PVDF/ZnO-NRs-15 and PVDF/ZnO-NSs-15 sensors were measured under mechanical loads ranging from 7 N to 27 N (Figure 7c,d,g,h). Generally, the output voltage and current were both linearly dependent on the applied force, as described by the piezoelectric constitutive relations.

The experimental results (Figure 7c,d) revealed that at a 7 N loading, the PVDF/ZnO-NRs-15 generated a peak-to-peak output voltage and current of 1.74 V and 21.04 nA, respectively. The PVDF/ZnO-NSs-15 exhibited superior performance, with a 2.10 V and 26.58 nA, as shown in Figure 7g,h. This trend of an enhanced response in the ZnO-NSs composites persisted across the tested force range, reaching maximum outputs of 3.84 V (PVDF/ZnO-NRs-15) and 4.75 V (PVDF/ZnO-NSs-15) at 27 N (Figure 7c,g). The enhanced outputs of the ZnO-NSs composites were due to the higher SSA, which amplified the interfacial polarization under mechanical deformation.

**Figure 8:**
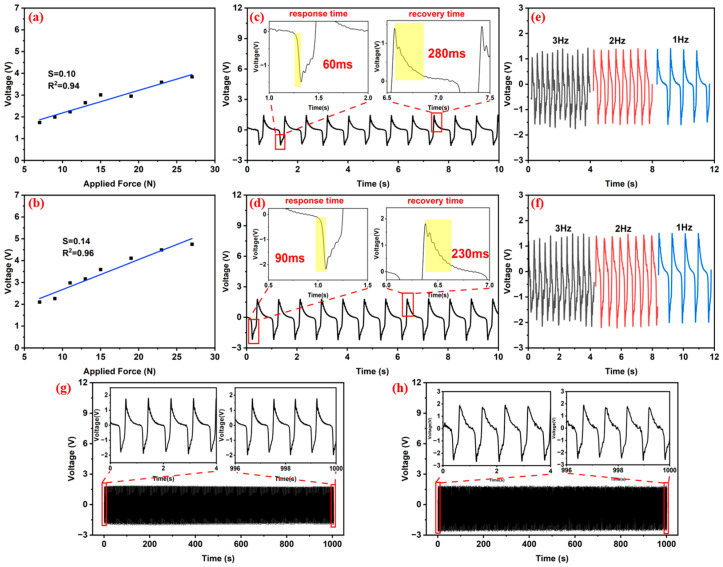
Output voltage vs. applied force for (**a**) PVDF/ZnO-NRs-15 and (**b**) PVDF/ZnO-NSs-15 sensor. Dynamic voltage curve for response time and recovery time for (**c**) PVDF/ZnO-NRs-15 and (**d**) PVDF/ZnO-NSs-15 sensor. Frequency response curve for (**e**) PVDF/ZnO-NRs-15 and (**f**) PVDF/ZnO-NSs-15 sensor. Fatigue test curve for (**g**) PVDF/ZnO-NRs-15 and (**h**) PVDF/ZnO-NSs-15 sensor.

The relationship between the applied force and electrical signal output was analyzed to evaluate the sensitivity of the pressure sensors. As illustrated in Figure 8, both the PVDF/ZnO-NRs-15 and PVDF/ZnO-NSs-15 sensors exhibited linear correlations between the electrical output and applied force, with the linear coefficients exceeding 0.9, indicating robust linear responses. Figure 8a reveals that the PVDF/ZnO-NRs-15 achieved a sensitivity of 100 mV/N. In comparison, the PVDF/ZnO-NSs-15 yielded an enhanced sensitivity of 140 mV/N, as displayed in Figure 8b, representing a 40% improvement over its counterpart. The ZnO-NSs nanostructure modification effectively enhanced the charge transfer efficiency in the composite system. The measured sensitivity values align with high-performance sensors’ characteristics, where sensitivity optimization typically involves the material selection and structural engineering. The outstanding sensitivity of the PVDF/ZnO-NSs-15 demonstrates its promising potential for applications requiring precise force detection (Table S1 in Supporting Information).

The response time and the recovery time were measured accordingly to assess the temporal response of the fabricated composite sensors. As shown in Figure 8c, under an external force of 25 N, the PVDF/ZnO-NRs-15 composite exhibited response and recovery times of 60 ms and 280 ms, respectively. As shown in Figure 8d, the PVDF/ZnO-NSs-15 composite demonstrated response and recovery times of 90 ms and 230 ms. In contrast, the PVDF/ZnO-NRs-15 achieved a shorter response time, but the PVDF/ZnO-NSs-15 had a faster recovery time. Both composites exhibited response times below 100 ms, highlighting their rapid responsiveness. This fast reaction speed suggests their promising potential for real-time monitoring applications in human motion detection.

A stepper motor was programmatically controlled to impact the sensor at varying frequencies (1 Hz, 2 Hz, and 3 Hz) by maintaining a constant applied force. Both the PVDF/ZnO-NRs-15 and PVDF/ZnO-NSs-15 sensors exhibited relatively stable output voltages under different impact frequencies (Figure 8e,f). The varying impact frequencies showed little influence on the output voltages.

The durability of the sensors, as a critical parameter for characterizing their long-term operational stability, was evaluated through a cyclic impact test with 1000 repetitions under an applied force of 22 N. After this 1000-cycle impact experiment, the peak-to-peak output voltage of the PVDF/ZnO-NRs-15 decreased from 3.86 V to 3.73 V (3.8% reduction) (Figure 8g), and that of the PVDF/ZnO-NSs-15 decreased from 4.54 V to 4.32 V (4.8% reduction) (Figure 8h). Both sensors demonstrated an output voltage degradation below the 5% threshold, confirming their signal stability and mechanical endurance during prolonged operation.

**Figure 9:**
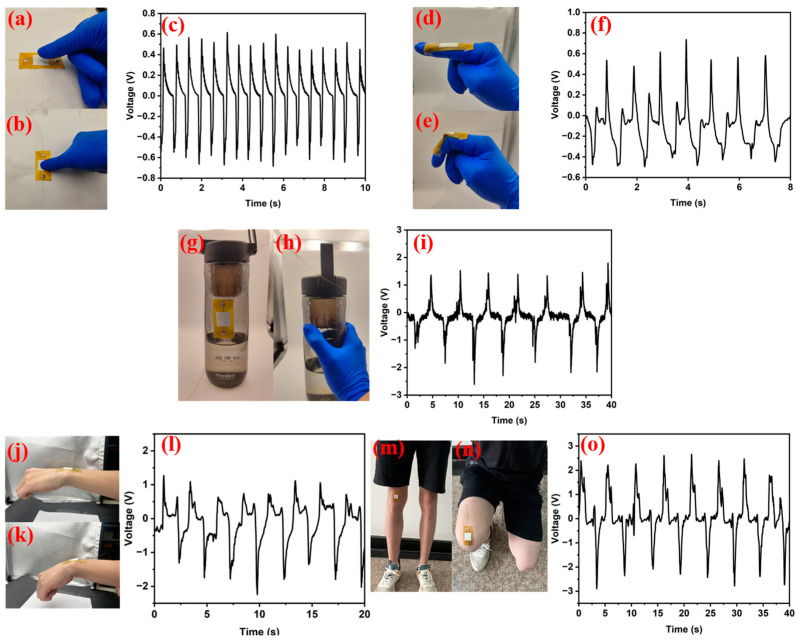
Sensing performance tests for the assembled sensors with the PVDF/ZnO-NS-15. A photo illustrating the (**a**) releasing and (**b**) pressing of a sensor mounted on a solid substrate, and the corresponding (**c**) voltage vs. time curve. A photo illustrating the (**d**) stretching and (**e**) bending of a sensor mounted on a finger, and the corresponding (**f**) voltage vs. time curve. A photo illustrating the (**g**) releasing and (**h**) holding of a sensor mounted on a plastic bottle, and the corresponding (**i**) voltage vs. time curve. A photo illustrating the (**j**) stretching and (**k**) bending of a sensor mounted on a wrist, and the corresponding (**l**) voltage vs. time curve. A photo illustrating the (**m**) stretching and (**n**) bending for a sensor mounted on a knee, and the corresponding (**o**) voltage vs. time curve.

The sensing performance of the composite sensor with the PVDF/ZnO-NSs on varied subjects was evaluated. The output voltage curves for the sensors are shown in Figure 9. For the case of flat-ground pressing in Figure 9a,b, when the finger applied pressure, the sensor generated a negative output voltage (Figure 9c). Upon releasing the pressure, a positive voltage was produced, followed by a rapid return to the baseline potential. As for the sensor mounted at a finger joint (Figure 9d,e), distinct piezoelectric signals were observed with joint flexion, yielding a peak-to-peak output voltage of 1.1 V (Figure 9f), which is pronounced to report or interpret finger motion.

The sensor was attached to a water cup. When the cup was grasped and subsequently released, the output voltage exhibited a dynamic response (Figure 9g,h). During continuous grasping and releasing, the voltage stabilized at equilibrium (Figure 9i). Upon an abrupt release, a reverse voltage spike emerged, reaching its peak before gradually returning to equilibrium. Further experiments evaluating the intense motions of a human wrist (Figure 9j–l) and knee (Figure 9m–o) were performed, yielding the characteristic patterns of piezoelectric output voltage response.

## 4. Discussion

The zeta-potential measurements demonstrated that the surface potentials of the ZnO-NRs and ZnO-NSs were −9.7 mV and −12.3 mV, respectively, confirming the negative charge of the particle surfaces. These negatively charged nanoparticles were then dispersed into PVDF matrices, serving as nucleation sites for the formation of the electroactive β-phase [27]. The strong electrostatic interactions between the CH_2_ dipoles in the PVDF chains and the negatively charged nanoparticles induced the alignment of the PVDF chains in a TTTT conformation at the nanoparticle interfaces, thereby facilitating β-phase crystallization [28]. Notably, the ZnO nanosheet held a more negative surface potential and a larger SSA compared to the rod-shaped ZnO, which synergistically enhanced the interfacial interactions and promoted a higher β-phase content in the PVDF.

The assembled sensor with the PVDF/ZnO-NSs composite film then generated a higher value of piezoelectric voltage in comparison with the one with the PVDF/ZnO-NRs composite. The morphology dependence of ZnO nanoparticles applied to piezoelectric sensors was thus assessed, indicating the favored shape of a nanosheet for the particular field of mechanical sensing. Future research should examine the particle morphology for inducing an elevated degree of β-phase content as well as the efficient production of composites.

## 5. Conclusions

In this study, two types of PVDF sensors incorporating ZnO nanoparticles with distinct morphologies were fabricated and evaluated. The sensor with the ZnO-NSs exhibited the highest output voltage at a content of 15 wt%, along with excellent sensitivity, frequency response characteristics, and fatigue resistance. This optimized configuration was subjected to a series of application-oriented tests, including mechanical durability assessments and performance evaluations, under simulated operational conditions. The developed sensor holds strong potential for applications in human motion detection.

## Data Availability

The data are contained within the article.

## Supplementary Materials

The following supporting information can be downloaded at: https://www.mdpi.com/article/10.3390/s25092936/s1, Table S1. Major parameters of recent composite PVDF/ZnO sensors. References [29,30,31,32] are cited in the Supplementary Materials.

## Abbreviations

The following abbreviations are used in this manuscript:

DIW	Direct Ink Writing
FTIR	Fourier-transform Infrared Spectroscopy
PVDF	Polyvinylidene Fluoride
SEM	Scanning Electron Microscopy
SSA	Specific Surface Area
XRD	X-ray diffraction
