# Supplementary material for: Morphology-Dependent Behavior of PVDF/ZnO Composites: Their Fabrication and Application in Pressure Sensors
Source: Sensors (Basel). 2025 May 7;25(9):2936. doi: 10.3390/s25092936 (PMC12074104; doi:10.3390/s25092936)
Supplement: Supplementary file 1 [file sensors-25-02936-s001.zip › sensors-3606992-supplementary.pdf]

Supporting Information

Morphology-dependent behavior of PVDF/ZnO Composites: Their Fabrication and Application in Pressure Sensors

Binbin Zhang [1,2], Wenhui Zhang [1], Wei Luo [3,*], Zhijie Liang [4], Yan Hong [1,4], Jianhui Li [5], Guoyun Zhou [1,*] and Wei He [1]

1 School of Materials and Energy, University of Electronic Science and Technology of China, Chengdu 611731, China; zhang0001234@163.com (B.Z.) 202221030230@std.uestc.edu.cn (W.Z.); he_wei@uestc.edu.cn (W.H.)
2 Beijing Spacecraft, China Academy of Space Technology, Beijing 100094, China
3 School of Integrated Circuits, Huazhong University of Science and Technology, Wuhan 430074, China
4 Jiangxi Institute of Electronic Circuit, Pingxiang 337009, China; liangzj0214@163.com
5 Suining Ruijiexing Technology Co., Ltd., Suining 629001, China; ljh@rjx-pcb.com
* Correspondence: luowei@hust.edu.cn (W.L.); gzhou32@uestc.edu.cn (G.Z.);

Table S1. Major parameters of recent composite PVDF/ZnO sensors.

Material type	Output Voltage	Sensitivity	Response time	References
PVDF/PZT	2.51 V/ 85.59 kPa	6.38 mV/N	21 ms	[1]
PVDF/ZnO	≈1.5 V/ 10 N	103 mV/N		[2]
PVDF	218.1 mV/ 2.83 N	78 mV/N		[3]
PVDF/PZT	184 mV/ 2.125 N	86.58 mV/N		[4]
PVDF/CNT	90 mV/ 350 N	2.26 mV/N		[5]
PVDF/TPU	≈3.3 V/ 20 N	95.8 mV/N	85 ms	[6]
PVDF/ZnO-NSs	3.56 V/ 15 N	140 mV/N	90 ms	this work

References

1. Tian, G.; Deng, W.; Gao, Y.; Xiong, D.; Yan, C.; He, X.; Yang, T.; Jin, L.; Chu, X.; Zhang, H.; et al. Rich lamellar crystal baklava-structured PZT/PVDF piezoelectric sensor toward individual table tennis training. *Nano Energy* **2019**, *59*, 574-581.
2. M, A.H.; Karumuthil, S.C.; Rajan, L. Optimization of PVDF nanocomposite based flexible piezoelectric tactile sensors: A comparative investigation. *Sensors and Actuators A: Physical* **2023**, *353*, 114215.
3. Yang, H.; Zhao, L.-z.; Zhang, Y.; Luo, H.; Wang, R.-c.; Zhang, D.; Wang, X.-f. Electrical properties of PVDF films fabricated by direct ink writing. *Journal of Central South University* **2023**, *30*, 1477-1489.
4. Chamankar, N.; Khajavi, R.; Yousefi, A.A.; Rashidi, A.; Golestanifard, F. A flexible piezoelectric pressure sensor based on PVDF nanocomposite fibers doped with PZT particles for energy harvesting applications. *Ceramics International* **2020**, *46*, 19669-19681.
5. Wu, C.-M.; Chou, M.-H.; Zeng, W.-Y. Piezoelectric Response of Aligned Electrospun Polyvinylidene Fluoride/Carbon Nanotube Nanofibrous Membranes. *Nanomaterials* **2018**, *8*.
6. Wei, C.; Zhou, H.; Zheng, B.; Zheng, H.; Shu, Q.; Du, H.; Ma, A.; Liu, H. Fully flexible and mechanically robust tactile sensors containing core–shell structured fibrous piezoelectric mat as sensitive layer. *Chemical Engineering Journal* **2023**, *476*, 146654.
